# “Decision tree analysis for assessing the risk of post-traumatic haemorrhage after mild traumatic brain injury in patients on oral anticoagulant therapy”

**DOI:** 10.1186/s12873-022-00610-y

**Published:** 2022-03-24

**Authors:** Gianni Turcato, Alessandro Cipriano, Naria Park, Arian Zaboli, Giorgio Ricci, Alessandro Riccardi, Greta Barbieri, Sara Gianpaoli, Grazia Guiddo, Massimo Santini, Norbert Pfeifer, Antonio Bonora, Ciro Paolillo, Roberto Lerza, Lorenzo Ghiadoni

**Affiliations:** 1 Emergency Department, Hospital of Merano (SABES-ASDAA), Via Rossini 5, 39012 Merano, Italy; 2 Emergency Department, Nuovo Santa Chiara Hospital, Azienda Ospedaliero-Universitaria Pisana, Pisa, Italy; 3 Emergency Department, University of Verona, Verona, Italy; 4 Academy of Emergency Medicine and Care (AcEMC), Pavia, Italy; 5 Emergency Department, Hospital of San Paolo (ASL N°2 Savonese), Savona, Italy; 6 Department of Clinical and Experimental Medicine, University of Pisa, Pisa, Italy

**Keywords:** Mild traumatic brain injury, Minor head injury, Risk factors, Oral anticoagulants, Anticoagulation, Emergency department, Decision tree, Machine learning, Minor head trauma, Trauma

## Abstract

**Background:**

The presence of oral anticoagulant therapy (OAT) alone, regardless of patient condition, is an indication for CT imaging in patients with mild traumatic brain injury (MTBI). Currently, no specific clinical decision rules are available for OAT patients. The aim of the study was to identify which clinical risk factors easily identifiable at first ED evaluation may be associated with an increased risk of post-traumatic intracranial haemorrhage (ICH) in OAT patients who suffered an MTBI.

**Methods:**

Three thousand fifty-four patients in OAT with MTBI from four Italian centers were retrospectively considered. A decision tree analysis using the classification and regression tree (CART) method was conducted to evaluate both the pre- and post-traumatic clinical risk factors most associated with the presence of post-traumatic ICH after MTBI and their possible role in determining the patient’s risk. The decision tree analysis used all clinical risk factors identified at the first ED evaluation as input predictor variables.

**Results:**

ICH following MTBI was present in 9.5% of patients (290/3054). The CART model created a decision tree using 5 risk factors, post-traumatic amnesia, post-traumatic transitory loss of consciousness, greater trauma dynamic, GCS less than 15, evidence of trauma above the clavicles, capable of stratifying patients into different increasing levels of ICH risk (from 2.5 to 61.4%). The absence of concussion and neurological alteration at admission appears to significantly reduce the possible presence of ICH.

**Conclusions:**

The machine-learning-based CART model identified distinct prognostic groups of patients with distinct outcomes according to on clinical risk factors. Decision trees can be useful as guidance in patient selection and risk stratification of patients in OAT with MTBI.

## Introduction

Intracranial haemorrhage (ICH) caused by mild traumatic brain injury (MTBI) is a relatively infrequent occurrence with a rate of life-saving neurosurgical interventions of less than 1% of cases [1–3]. Currently, the diagnostic gold standard for identifying post-traumatic ICH remains head CT, and despite attempts to limit its extensive use in recent years, CT prescriptions after MTBI have increased exponentially [4]. The possible negative implications for the patient, but also for the managing physician, caused by a failure to detect post-traumatic hemorrhage, added to the increase in ED accesses even for minor trauma, have led to the almost routine use of CT in patients with MTBI in clinical practice, regardless of the real haemorrhagic risk presented [4, 5]. The concomitant use of oral anticoagulant therapy (OAT), known to promote the development of post-traumatic ICH, has led many international guidelines to indicate that at least one head CT should be performed in all patients with MTBI on OAT regardless of the extent of trauma and the clinical condition presented by the patient in the ED [6]. A high number of negative examinations, increased costs, and lengthier stay in the ED are just some of the consequences of unreasoned use of CT even in patients with OAT [3, 7]. At present, there is no specific decision rule dedicated only to OAT patients with MTBI; moreover, it seems that the presence of the anticoagulation alone is considered sufficient to impose head imaging [8]. Recent evidence seems to suggest that clinical risk factors, already extensively studied in traumatic brain injury for defining the risk of bleeding before head CT, could also be used in the first assessment of the patient with OAT [8–10]. The confirmation of a specific role of clinical risk factors, also in patients on OAT could allow the creation of specific workups for these patients, identifying patients with certain risk factors that need to be investigated with CT, and to safely exclude the other patients.

The classification and regression tree (CART) analysis is a machine learning technique that allows the design of a decision tree to support decision-making processes [11, 12]. Recently, breakdown techniques in data mining, of which CART analysis is a part, have been used in many fields of medicine for the development of predictions and the interpretation of the relationship between variables. Unlike older logistic regressions, machine learning can identify random relationships that may not be evident with other techniques. Also, machine learning has better efficiency and accuracy, and the models which are based on this approach generally have better predictive predictions [11, 12].

The current study, developing a hierarchical prognostic model using the powerful CART methodology, aims to confirm the role of some clinical and laboratory factors in assessing the risk of post-traumatic ICH after MTBI in patients with OAT [11, 12].

## Methods

### Study design and setting

The present study is a multicentre retrospective observational study. It involved the Emergency Departments (ED) of four Italian centers: the Hospital Civile Maggiore of Verona (Italy 100,000 visits per year), the University Hospital of Verona (Italy 50,000 visits per year), the University Hospital of Pisa (Italy 90,000 visits per year) and the General Hospital of Merano (Italy 70,000 visits per year). The study was conducted with the approval of the local ethics committees (Ethics Committee for Clinical Trials, Verona, Italy, approval number 889CESC; Ethics Committee for Clinical Trials, Bolzano, Italy, approval number 75-2019; Ethics Committee for Clinical Trials, Pisa, Italy 11924_CIPRIANO) and was conducted according to the ethical principles for medical research involving human subjects of the Declaration of Helsinki.

### Patients

All patients in OAT who required an evaluation in the ED for an MTBI between 1 January 2016 and 31 December 2019, were considered. MTBI was considered as any closed trauma of the cranio-facial district associated with a Glasgow Coma Scale (GCS) of 14-15 at presentation and regardless of loss of consciousness immediately following the trauma [6, 13, 14]. Exclusion criteria were: having access to the ED more than 48 hours after trauma, ineffective OAT, being defined as inadequate intake of Vitamin K Antagonist (VKA) for more than 1 week before the trauma, or having direct oral anticoagulants (DOACs) intake no less than 24 hours before the trauma, having inadequate anticoagulation with VKA, which was defined as International Normalized Ratio (INR) < 1.5.

The records of patients treated with OAT and MTBI were identified according to the following procedure. All patients who underwent cranial CT in the ED during the study period were extracted from the respective computer databases, using dedicated management software (FirstSTATA for Verona and Pisa and QlikView for Merano). The selection of OAT patients only, the congruence with the definition of MTBI, the exclusion criteria, and the recording of baseline and study characteristics were performed with a manual chart review by a group of emergency physicians with more than 5 years of experience.

### Clinical management of patients with mild traumatic brain injury

Since 2014, the hospitals under study follow a management protocol for patients with MTBI, based on national guidelines [15]. For patients admitted to the ED for an MTBI in OAT, a head CT is performed on admission and an observation period of not less than 24-h is recommended with the possibility of performing a second head CT before discharge. The protocol also included the collection of pre- and post-traumatic risk factors. Pre-trauma factors were: age ≥ 65 years, presence of antiplatelet therapy, alcohol or drug intoxication on ED arrival, dementia or major psychiatric problems, history of epilepsy, and previous history of neurosurgery. Post-traumatic factors were: major trauma dynamics (defined as a high-speed road traffic accident either as a pedestrian/cyclist or vehicle occupant), fall from a height of more than 3 m, high-speed object accident, post-traumatic transitory loss of consciousness (TLOC), post-traumatic amnesia (any type of post-traumatic amnesia has been considered), presence of post-traumatic headache, presence of signs of trauma above the clavicles, clinical signs of skull base fracture, at least one episode of vomiting, post-traumatic seizure, post-traumatic neurological deficit and a GCS < 15 [6, 15]. The presence or absence of these factors during the patient evaluation was recorded in the ED’s medical chart.

### Outcomes

Finding of post-traumatic ICH in head CT scans performed on arrival in the ED (immediate), or in head CT scans performed after 24 h of clinical observation (delayed) was the primary endpoint of the study. CT scan positivity was considered as the presence of subdural, epidural, or parenchymal haematoma, subarachnoid haemorrhage or cerebral contusion [13, 14, 16]. Finally, important patient outcomes were defined as the need for neurosurgical intervention (craniotomy, craniectomy, placement of a hole or subdural drainage) or death from post-traumatic ICH within 30 days of trauma [8, 9, 13, 14, 16]. Patient follow-up was reconstructed by evaluating the medical records available in the computer databases of the EDs in the study, and mortality was confirmed through the registry office.

### Decision tree

Decision tree analyses are powerful data mining techniques that are used to classify a set of data and obtain predictions about a dependent output variable. They produce classification and prediction models that help in many decision-making processes. The algorithm underlying the decision trees involves the hierarchical division of the population data into homogeneous subsets according to precise splitting rules. The breakdown of the data, obtained through a set of independent input variables, allows predictions to be made about the target-dependent variable. The decision tree analysis produces a hierarchical diagram consisting of a set of elements called nodes. The node from which subsequent nodes branch is called the root and is composed of the most significant independent variable. Subsequent levels are composed of parent nodes that identify levels and are followed by other nodes at lower levels. Terminal nodes, those that are not further subdivided into other nodes, are also called leaf nodes and can identify subgroups of patients sharing the same risk conditions. The diagram provided by the decision trees is easy to interpret, allows an immediate intuition of the effect of an independent variable on the dependent variable, and, unlike previous regression models, allows an easy interpretation of the relationship between all the variables of the model.

### Statistical analysis

Categorical variables were described as percentage and number of events in the total while continuous variables were described as mean and standard deviation (SD) or as the median and interquartile range (IRQ) depending on the underlying distribution. Univariate comparisons were performed with Fischer’s Exact test, Chi-square test, Mann-Whitney test, and Kruskas-Wallis test. Variables found to be significant in the previous univariate analysis (*p* < 0.05) were entered into the multivariate model. Where appropriate, the Tukey transformation was used to re-express the continuous variables entered in the model, using a power transformation, where the assumption of normality was not guaranteed. A binary logistic regression was used for the multivariate model using the stepwise regression method. Odd ratios with 95% confidence intervals were reported. The study constructed a decision tree using the CART technique. The CART model, a machine-learning and data-mining recursive algorithm were used to identify groups of patients with a homogeneous risk of post-traumatic ICH and to study the hierarchical association between clinical and laboratory risk factors.

Statistical analyses were performed with STATA 16.1 statistical software (StataCorp, College Station, TX, USA).

## Results

### Populations

**Fig. 1:**
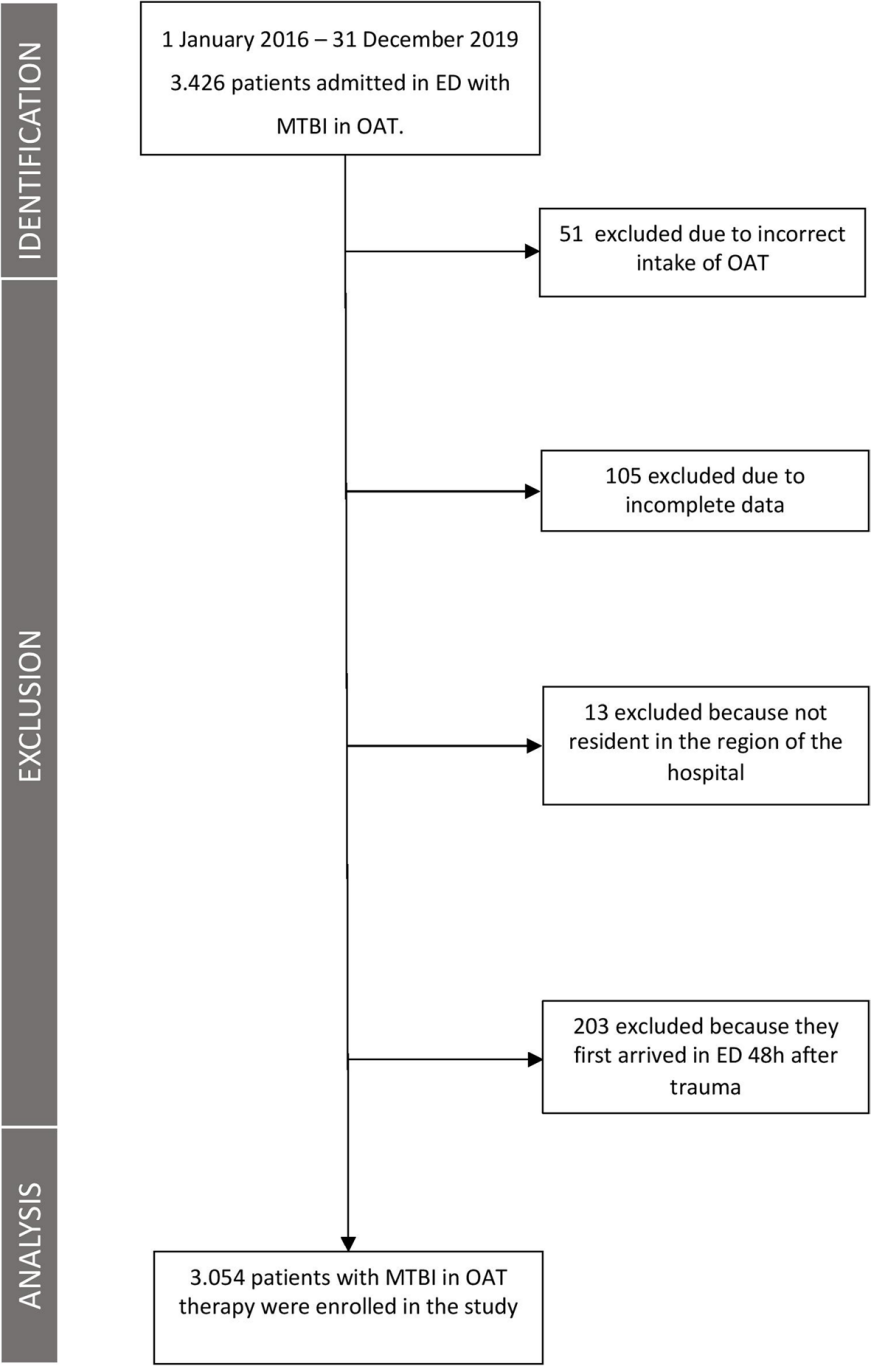
Flow-chart of patients enrolled in the study

The number of OAT patients who underwent at least one CT scan for an MTBI was 3054 (Fig. 1). The baseline characteristics of the patients are described in Table 1.

**Table 1:** Baseline characteristics of the cohort of patients with MTBI and on OAT undergoing CT in the ED.

**Patients, n (%)**	3054 (100)
**Age in years, median (IQR)**	83 (77-88)
**Sex, n (%)**	
Male	1414 (46.3)
Female	1640 (53.7)
**Oral anticoagulant therapy, n (%)**	
DOACs	1212 (39.7)
VKA	1842 (60.3)
**Reason for Oral anticoagulant therapy intake, n (%)**	
Atrial fibrillation	2673 (87.5)
Pulmonary embolism	196 (6.4)
Heart valves	70 (2.3)
Others	115 (3.8)
**Mechanism of trauma, n (%)**	
Accidental falls	2450 (80.2)
Road incident	155 (5.1)
Syncope or epilepsy	198 (13.0)
Direct trauma	51 (1.7)
**Outcome, n (%)**	
Global ICH	290 (9.5)
Immediate ICH	253 (8.3)
Delayed ICH	37 (1.2)
Neurosurgery or death at 30 days for ICH	43 (1.4)

The median age of patients enrolled in the study was 83 years (IQR 77-88) and they were predominantly female. 60.3% of patients (1842/3054) were on VKA therapy while 39.7% (1212/3054) were on DOACs. The main reason for the need for OAT was atrial fibrillation and the main mechanism of injury was an accidental fall. Overall, post-traumatic ICH occurred in 9.5% of patients (290/3054) on OAT. The 1.4% (43/3054) of patients underwent neurosurgery or died within 30 days as a result of ICH.

### Analysis of clinical risk factors for ICH

The clinical factors recorded at the time of the first ED evaluation are reported in Table 2.

**Table 2:** Univariate analysis of pre-traumatic and post-traumatic risk factors with the presence of post-traumatic ICH in patients receiving OAT

Variable	No ICH	ICH	***p***-value
**Patients, n (%)**	2764 (90.5)	290 (9.5)	
**Age in years, median (IQR)**	83 (77-87)	84 (78-88)	0.030
**Sex, n (%)**			0.155
Male	1268 (45.9)	146 (50.3)	
Female	1496 (54.1)	144 (49.7)	
**Oral anticoagulant therapy, n (%)**			0.002
VKA	1642 (59.4)	200 (69)	
DOACs	1122 (40.6)	90 (31)	
**Pre-traumatic risk factors, n (%)**			
Major trauma dynamic	94 (3.4)	42 (14.4)	< 0.001
Acute intoxication	59 (2.1)	6 (2.1)	1.000
Previous neurosurgery	66 (2.4)	20 (6.9)	< 0.001
Antiplatelet therapy	125 (4.5)	23 (7.9)	0.014
**Post-traumatic risk factors, n (%)**			
Post-traumatic TLOC	112 (4.1)	60 (20.7)	< 0.001
Post-traumatic amnesia	208 (7.5)	89 (30.7)	< 0.001
Post-traumatic headache	85 (3.1)	27 (9.3)	< 0.001
GCS < 15	277 (10.0)	88 (30.3)	< 0.001
Focal neurological signs	23 (0.8)	15 (5.2)	< 0.001
Visibile trauma above the clavicles	1780 (64.4)	243 (83.8)	< 0.001
Post-traumatic vomiting	53 (1.9)	31 (10.7)	< 0.001
Post-traumatic seizure	6 (0.2)	3 (1.0)	0.046

Sixty-nine percent (200/290) of patients who presented with post-traumatic ICH were on VKA while only 31% (90/290) were taking DOACs, *p* = 0.002.

Among pre-trauma risk factors, those associated with post-traumatic ICH had: greater trauma dynamics (14.4% vs. 3.4%, *p* < 0.001), previous history of neurosurgery (6.9% vs. 2.4%, *p* < 0.001) and concomitant antiplatelet therapy (7.9% vs. 4.5%, *p* = 0.014).

Post-traumatic risk factors associated with the presence of post-traumatic ICH had: a post-traumatic TLOC (20.7% vs. 4.1%, *p* < 0.001), post-traumatic amnesia (30.7% vs. 7.5%, *p* < 0.001), a post-traumatic headache (9.3% vs. 3.1%, *p* < 0.001), a GCS of less than 15 at the ED examination (30. 3% vs. 10%, *p* < 0.001), the presence of a focal neurological deficit (5.2% vs. 0.8%, *p* < 0.001), evidence of trauma above the clavicles (83.8% vs. 64.4%, *p* < 0.001), post-traumatic vomiting (10.7% vs. 1.9%, *p* < 0.001) and post-traumatic seizure (1.0% vs. 0.2%, *p* = 0.047) (Table 2).

None of the vital parameters recorded at admission were associated with the risk of post-traumatic ICH. Among the main laboratory parameters performed in the evaluation of patients with MTBI and OAT, those associated with the presence of post-traumatic ICH were platelet count (196 vs. 206, *p* = 0.022) and blood glucose (7.1 vs. 6.6, *p* = 0.005) (Table 3).

**Table 3:** Univariate analysis between risk of post-traumatic ICH, vital parameters and the main blood samples in patients with MTBI in OAT

Variable	No ICH	ICH	***p***-value
**Patients, n (%)**	2764 (90.5)	290 (9.5)	
**Vital parameters**			
Systolic BP (mmHg), mean (SD)	147 (26)	148 (28)	0.395
Diastolic BP (mmHg), mean (SD)	78 (13)	78 (14)	0.296
HR (bpm), median (IQR)	78 (69-89)	78 (69-90)	0.474
Oxygen saturation (%), median (IQR)	96 (95-98)	96 (94-98)	0.642
**Laboratory parameters**			
Haemoglobin (g/dL), mean (SD)	126 (18)	126 (19)	0.660
Platelets (× 1000/ μL), median (IQR)	206 (168-253)	196 (159-246)	0.022
Blood sugar (mmol/L), median (IQR)	6.6 (5.7-8.2)	7.1 (5.8-8.7)	0.005
Creatinine (mmol/L), median (IQR)	91 (73-122)	88 (73-112)	0.832
Protrombine time (INR), median (IQR)	1.99 (1.42-2.64)	2.07 (1.5-2.71)	0.227
Activated partial thromboplastin time (ratio), median (IQR)	1,14 (1.01-1.31)	1.14 (1.01-1.34)	0.864

### Multivariate analysis

Using binary logistic regression, a multivariate model to assess the risk of post-traumatic ICH (dependent variable) was produced by including clinical and laboratory variables found to be significant in the previous univariate analysis (Table 4).

**Table 4:** Multivariate analysis using backward regression method between risk factors found to be associated with the risk of post-traumatic ICH in the previous univariate analysis

Variable	Coefficient	Error	OR	CI95%	p-value
Major trauma dynamic	0.829	0.245	2.290	1.418-3.698	0.001
Previous neurosurgery	1.285	0.290	3.613	2.046-6.382	< 0.001
Post-traumatic TLOC	1.035	0.215	2.816	1.847-4.294	< 0.001
Post-traumatic amnesia	1.026	0.176	2.789	1.974-3.940	< 0.001
Headache	0.843	0.283	2.324	1.334-4.047	0.003
GCS < 15	1.117	0.164	3.056	2.216-4.213	< 0.001
Visibile trauma above the clavicle	0.982	0.172	2.669	1.907-3.735	< 0.001
Focal neurological signs	1.523	0.394	4.587	2.119-9.932	< 0.001
Post-traumatic vomiting	1.041	0.292	2.833	1.597-5.025	< 0.001

None of the laboratory parameters were found to be independent risk factors for ICH. Nine clinical risk factors were found to be predictive of post-traumatic ICH (Table 4). These risk factors are: major trauma dynamic (OR 2.290, CI95% 1.418-3.698, *p* = 0.001), previous neurosurgical intervention (OR 3.613, CI95% 2.046-6.382, *p* < 0. 001), post-traumatic TLOC (OR 2.816, CI95% 1.847-4.294, < 0.001), post-traumatic amnesia (OR 2.789, CI95% 1.974-3.940 < 0.00), post-traumatic headache (OR 2. 324, CI95% 1.334-4.047, *p* = 0.003), a GCS of less than 15 (OR 3.056, CI95% 2.216-4.213 < 0.001), evidence of trauma above the clavicles (OR 2.669, CI95% 1.907-3.735, < 0.001), a focal neurological sign (OR 4.587, CI95% 2.119-9.932, < 0.001) and the presence of post-traumatic vomiting (OR 2.833, CI95% 1.597-5.025, < 0.001).

### CART-model

**Fig. 2:**
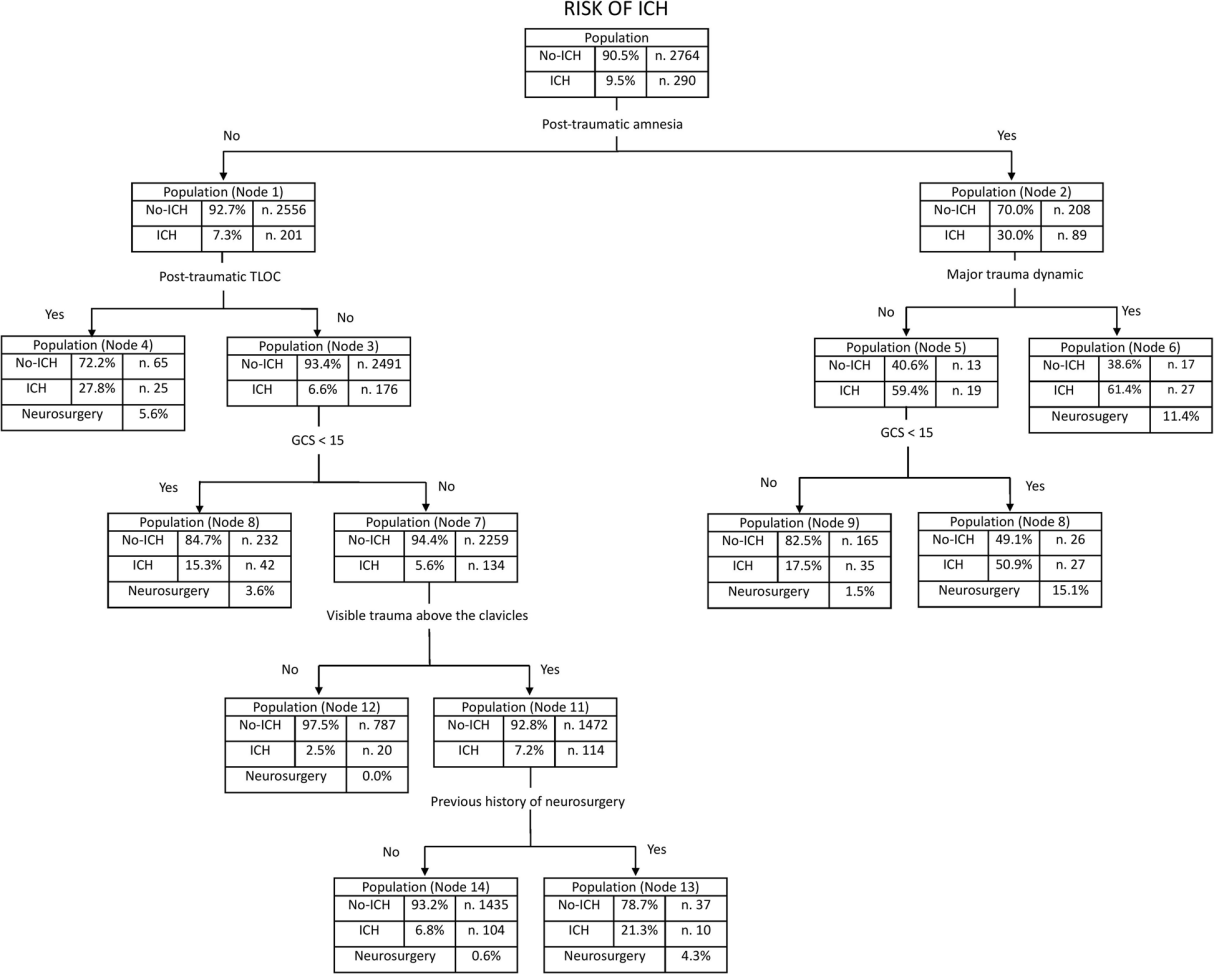
Decision tree model generated using the chi-square automatic interaction detection (CHAID) method that represents the hierarchical association of pre- and post-traumatic risk factors related to the presence of post-traumatic intracranial haemorrhage in patients taking oral anticoagulants. The rate of severe outcomes for each “leaf” node is reported

Of all the clinical, parametric, and laboratory variables, the decision tree analysis identified seven significant and important variables in the evaluation of the patient in OAT with MTBI (Fig. 2). No laboratory or clinical variables were found to be significant in the decision tree. The most important predictor, which formed the root node, was post-traumatic amnesia. The other predictors were: post-traumatic TLOC, major trauma dynamics, a GCS of less than 15, and evidence of trauma above the clavicles. The decision tree identified 8 leaf nodes with a probability of ICH risk ranging from 2.5 to 61.4%. Positivity of the root node (post-traumatic amnesia) or its most important child node (node 1, post-traumatic TLOC) carries a high risk of post-traumatic ICH ranging from 17.5% (Node 9) to 61.4% (Node 6). Root node negativity, combined with the exclusion of post-traumatic TLOC (Node 1, risk 27.8%) and a negative neurological condition at the time of the examination (GCS not less than 15, Node 3), identifies a more limited risk of ICH, generally less than 10% (Nodes 12 and 14). Progressive exclusion of risk factors along the flow-chart reduces both the risk of ICH and the risk of a serious outcome (Fig. 2). The decision tree shows a 98.4% correctness rate for exclusion of post-traumatic ICH with an overall risk of error close to 9%. Exclusion of all clinical factors in the tree (Node 12) or the mere presence of evidence of trauma above the clavicles (Node 14) indicate very low-risk profiles for the need for neurosurgical intervention (Node 12 0%, Node 14 0.6%).

## Discussion

Using the largest cohort of patients on OAT and exploiting the potential of machine learning for the first time, the present study evaluated the clinical role of known risk factors in predicting post-traumatic hemorrhage after MTBI in OAT patients. In the last decades, the role of clinical risk factors in traumatic brain injury has been extensively studied, and many of these have been incorporated into clinical decision rules that assist the clinician in the assessment of the patient’s risk of bleeding after MTBI [2, 14]. Although some first indications have recently been published about the possible role of some clinical and laboratory factors also in anticoagulated patients, no solid and confirmed evidence is currently available about the possible role of these predictive factors in determining the risk of ICH in patients with OAT [8–10, 17, 18]. Such consolidation seems to be indispensable to subsequently propose a standardized and precise assessment for the stratification of clinical risk of ICH in this particular population [8–10, 18]. The lack of information about the possible predictive role of clinical risk factors has led the main guidelines, although not supported by evidence but by expert opinions only, to recommend the performance of a cranial CT scan in all OAT patients indiscriminately from their presentation [6, 15, 16, 18]. The CART model provided a hierarchical weighting of the different prognostic factors to identify which factors at the first ED evaluation had the greatest prognostic impact. Specifically, the CART model identified post-traumatic TLOC, major dynamic, a GCS < 15, evidence of trauma above the clavicles, and a previous history of neurosurgery as the most important factors to consider for ICH risk in patients with MTBI in OAT, results that are consistent with previous early evidence [8, 9].

The incidence of post-traumatic ICH in OAT patients with MTBI, is reported between 1 and 8%, in the studies published in recent years, with a significant difference between VKA and DOACs [10, 17, 19, 20].

Apparently, this limited incidence of post-traumatic ICH, also comparable to the cohort of the present study, does not seem to justify an extensive and unreasoned use of cranial CT in all patients with MTBI and OAT [10, 17, 19–21]. The low rates of need for neurosurgical intervention or trauma-related death seem to confirm that a preliminary assessment of the patient for imaging can be considered even during OAT [16, 19]. The real contribution of OAT to the risk of bleeding in patients with MTBI is, in fact, not yet fully understood. According to Uccella et al., the OAT patient had a risk of death comparable to the population without OAT, while Wiegele et al. highlighted the increased risk of ICH from OAT [22, 23]. However, according to Uccella et al., the risk of death from OAT was lower than in patients on antiplatelet therapy [22]. Recently, Moore et al. also reported an intermediate risk for patients on OAT between patients without hemostatic modifying therapy and patients on antiplatelet therapy [24]. There is no doubt that when the presence of an OAT is analyzed as a single predictor variable within a cohort of patients with or without anticoagulation, it is an independent predictor of ICH risk with an OR of 2.7 [25]. However, this contribution to patient risk appears to be similar to that of other clinical variables (e.g. TLOC, major dynamics) which do not currently induce the need for imaging in the patient without anticoagulation [6, 26]. It would seem possible, therefore, not to consider the presence of anticoagulant therapy as the only discriminator for the use of head CT, but rather as an important factor to be considered within a broader analysis of post-traumatic haemorrhagic risk in patients with MTBI.

Similar to the results of the present study, some recent evidence seems to suggest that despite the presence of OAT, the absence of any other clinical risk factor reduces the likelihood of post-traumatic ICH. In their study, Galliazzo et al. reported that pre-and post-traumatic clinical risk factors are important in predicting the risk of ICH and, in particular, that OAT alone is not associated with an independent increase in the risk of developing ICH in patients with MTBI [27]. Fuller et al. indicated that the absence of neurological alterations (GCS 15) and the absence of major trauma dynamics ruled out the presence of any complications at CT, identifying the OAT risk factor as an insufficient predictor unless combined with other clinical and laboratory findings [18]. If we consider the clinical implications of these ICH detected at head CT, the need for intervention or important outcomes becomes even rarer if there are no risk factors other than OAT [18, 19, 22]. It is therefore possible that, although the concomitant presence of OAT, the sequential exclusion of the other risk factors leads to a progressive reduction in risk, as demonstrated by decision tree analysis. Conceptually, the current view of the clinical work in the ED of patients with MTBI and OAT could be reversed by shifting from the current management focused on the exhausting search for confirmation of a possible ICH, which although apparently safer is burdened by very high rates of negative head CT, to a clinical work of exclusion, where through a reasoned and sequential use of established risk factors the ED physician excludes lower-risk patients limiting the use of CT to selected higher-risk patients although it was not the intention of the current study to suggest any conclusive procedures for the management of the patient with OAT and MTBI, the analysis performed can suggest, as previously demonstrated by the Canadian CT Head Rule, that a workup based on multistep rule out processes can also be designed and validated in OAT patients [2]. Further, adequately powered prospective studies are needed to demonstrate that this approach can be effective and to create a tool applicable in the real clinical practice, however this appears to be the first study to advance a new way of dealing with MTBI in OAT patients.

The study presents some limitations. First, the retrospective nature of the study exposes it to the possible biases inherent in this study model. The presence of a large number of patients, the multiple centers involved and the presence of clinical protocols focused on the careful assessment of risk factors may have greatly reduced the impact of possible biases. Secondly, as there is no unanimous definition, the current study used the same definition of MTBI as the most recent studies on MTBI and OAT [8, 9, 16]. Third, no cross-validation methodologies were used to identify the decision tree. Fourth, a proportion of patients did not repeat head CT after a first negative CT but were discharged after clinical observation, as reported in the clinical protocol, these patients were considered negative for ICH considering the first negative CT.

## Conclusions

The risk assessment of post-traumatic ICH in patients with OAT and an MTBI proposed by the guidelines is based only on head CT imaging, regardless of the patient’s clinical presentation. The low incidence rates of post-traumatic ICH, which are comparable to the non-anticoagulated population, combined with minimal risk of requiring life-saving neurosurgical intervention, seem not to justify the current extensive and routine use of CT. Through the powerful and innovative analysis of decision trees, it was confirmed that the assessment of clinical risk factors can help predict the risk of post-traumatic ICH also in OAT patients. The machine-based, CART model could provide an easy-to-interpret representation of variables associated with the risk of post-traumatic ICH and could be used as a guide for patient selection and risk stratification.

## Data Availability

The datasets used and analysed during the current study are available from the corresponding author on reasonable request.

## Abbreviations

MTBI: Mild traumatic brain injury
ED: Emergency department
CT: Computer tomography
OAT: Oral anticoagulant therapy
ICH: Intracranial haemorrhage
GCS: Glasgow coma scale
VKA: Vitamin K antagonist
DOACs: Direct oral anticoagulants
TLOC: Transitory loss of consciousness
CART: Classification and regression tree
